# Dioxygen Reactivity of Copper(I)/Manganese(II)-Porphyrin Assemblies: Mechanistic Studies and Cooperative Activation of O_2_

**DOI:** 10.3390/molecules27031000

**Published:** 2022-02-01

**Authors:** Runzi Li, Firoz Shah Tuglak Khan, Shabnam Hematian

**Affiliations:** Department of Chemistry and Biochemistry, University of North Carolina at Greensboro, Greensboro, NC 27412, USA; r_li4@uncg.edu (R.L.); fskhan@uncg.edu (F.S.T.K.)

**Keywords:** manganese porphyrins, copper chelates, dioxygen chemistry, variable-temperature absorption spectroscopy, nuclear magnetic resonance spectroscopy

## Abstract

The oxidation of transition metals such as manganese and copper by dioxygen (O_2_) is of great interest to chemists and biochemists for fundamental and practical reasons. In this report, the O_2_ reactivities of 1:1 and 1:2 mixtures of [(TPP)Mn^II^] (**1**; TPP: Tetraphenylporphyrin) and [(tmpa)Cu^I^(MeCN)]^+^ (**2**; TMPA: Tris(2-pyridylmethyl)amine) in 2-methyltetrahydrofuran (MeTHF) are described. Variable-temperature (−110 °C to room temperature) absorption spectroscopic measurements support that, at low temperature, oxygenation of the (TPP)Mn/Cu mixtures leads to rapid formation of a cupric superoxo intermediate, [(tmpa)Cu^II^(O_2_^•–^)]^+^ (**3**), independent of the presence of the manganese porphyrin complex (**1**). Complex **3** subsequently reacts with **1** to form a heterobinuclear *μ*-peroxo species, [(tmpa)Cu^II^–(O_2_^2–^)–Mn^III^(TPP)]^+^ (**4**; *λ*_max_ = 443 nm), which thermally converts to a *μ*-oxo complex, [(tmpa)Cu^II^–O–Mn^III^(TPP)]^+^ (**5**; *λ*_max_ = 434 and 466 nm), confirmed by electrospray ionization mass spectrometry and nuclear magnetic resonance spectroscopy. In the 1:2 (TPP)Mn/Cu mixture, **4** is subsequently attacked by a second equivalent of **3**, giving a *bis*-*μ*-peroxo species, i.e., [(tmpa)Cu^II^−(O_2_^2−^)−Mn^IV^(TPP)−(O_2_^2−^)−Cu^II^(tmpa)]^2+^ (**7**; *λ*_max_ = 420 nm and δ_pyrrolic_ = −44.90 ppm). The final decomposition product of the (TPP)Mn/Cu/O_2_ chemistry in MeTHF is [(TPP)Mn^III^(MeTHF)_2_]^+^ (**6**), whose X-ray structure is also presented and compared to literature analogs.

## 1. Introduction

Dioxygen (O_2_) binding, reduction, and activation at metalloenzyme active sites are vital for aerobic life. Manganese-containing enzymes, in particular, facilitate a wide variety of biological redox processes through interactions of manganese with O_2_ and its reduced derivatives such as superoxide (O_2_^•–^) and peroxide (O_2_^2–^) [1,2,3]. Because of its relative abundance and multiple accessible oxidation states, manganese can facilitate biochemical multielectron redox conversions and is essential for a variety of biological redox processes such as photosynthetic O_2_ evolution, antioxidant defense mechanisms regulating reactive oxygen species (ROS), and DNA synthesis [1,2,3,4]. Several Mn-containing enzymes involved in facilitating these redox processes include manganese superoxide dismutase (SOD), manganese catalase, the oxygen-evolving complex (OEC) in photosystem II, and Mn/Mn or Mn/Fe ribonucleotide reductase (RNR) [2,3,5]. The nuclearity of such manganese active sites varies, with more complex redox processes typically occurring at multinuclear sites with two or more metal centers [6,7].

Over the years, many Mn-porphyrins have been investigated for their ability to mimic various enzymatic activities or have been used as catalysts to promote a variety of other redox reactions [1,8,9,10,11,12,13,14,15,16,17,18,19,20,21]. An extensively-studied example is tetraphenylporphyrin manganese(II), (TPP)Mn^II^, which was used for generating the first dioxygen adduct of a manganese-containing complex [22]. While no reactivity toward O_2_ was observed at room temperature, it was found that the complex can be reversibly oxygenated at cryogenic conditions (<−80 °C), forming a manganese(IV) peroxide species, i.e., (TPP)Mn^IV^O_2_^2–^ [22,23]. This reactivity significantly differs from those observed for iron(II) or cobalt(II) analogues, which typically produce “superoxide-like” adducts [24,25]. Later, Valentine and co-workers reported the formation of a manganese(III) peroxide adduct, (TPP)Mn^III^O_2_^2–^, through the reaction of equimolar quantities of potassium superoxide (KO_2_) and (TPP)Mn^II^ [24,26]. However, the addition of more than one equivalent of the superoxide ion was not reported.

Although O_2_ is a very powerful four-electron oxidant, its one-electron reduction to generate superoxide as the product is relatively disfavored. Instead, metal-O_2_ reactivity typically requires the presence of protons or proceeds through a two-electron step to form a peroxide adduct. The two-electron step may involve O_2_ obtaining two electrons from one metal or from two discrete metal centers forming a binuclear *µ*-peroxo complex [26,27].

Due to the versatility of manganese-O_2_ chemistry, the catalytic advantage of multinuclear sites, and the undeniably rich chemistry of copper with dioxygen, in this study, we present the oxygenation chemistry of 1:1 and 1:2 mixtures of a copper(I)/manganese(II)-porphyrin system, i.e., [(TPP)Mn^II^] (**1**) and [(tmpa)Cu^I^(MeCN)][B(C_6_F_5_)_4_] (**2**), where TMPA is tris(2-pyridylmethyl)amine.

**Scheme 1:**
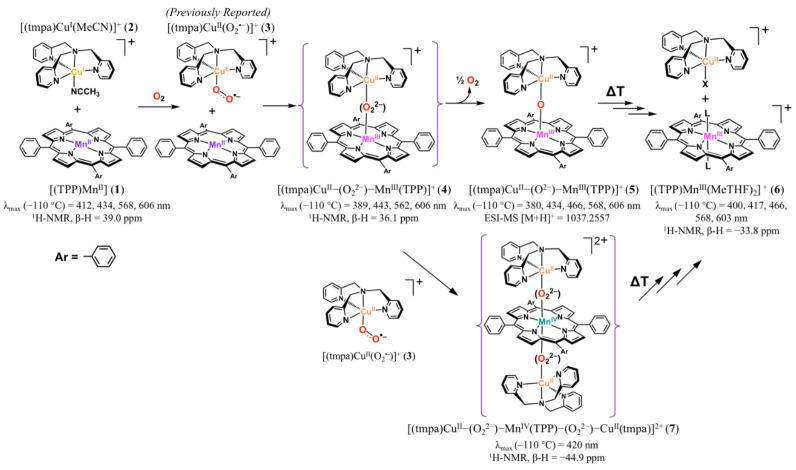
Proposed reaction steps for the oxygenation of 1:1 and 1:2 mixtures of [(TPP)Mn^II^] (**1**) and [(tmpa)Cu^I^(MeCN)][B(C_6_F_5_)_4_] (**2**) in MeTHF leading to the observed products. TPP: Tetraphenylporphyrin; TMPA: Tris(2-pyridylmethyl)amine).

A very rich chemistry ensued (Scheme 1), wherein there was an initial rapid generation of a cupric-superoxo complex, [(tmpa)Cu^II^(O_2_^•–^)]^+^ (**3**), as described previously [27,28,29,30,31]. In the reaction mixture, the cupric-superoxo species subsequently reacted with **1**, forming a heterobinuclear O_2_-adduct formulated as the *μ*-peroxo [(tmpa)Cu^II^–(O_2_^2–^)–Mn^III^(TPP)]^+^ (**4**) assembly. In the 1:2 mixture, this was followed by the subsequent addition of a second equivalent of **3** to **4**, forming a *bis*-*μ*-peroxo [(tmpa)Cu^II^−(O_2_^2−^)−Mn^IV^(TPP)−(O_2_^2−^)−Cu^II^(tmpa)]^2+^ (**7**) species. These two dioxygen-adducts have been characterized by ultraviolet–visible (UV-vis) absorption and nuclear magnetic resonance (NMR) spectroscopies. These bridged peroxo assemblies (i.e., **4** and **7**) thermally transform to the corresponding *μ*-oxo and possibly *bis*-*μ*-oxo complexes, respectively, which, in turn, decompose to [(TPP)Mn^III^(MeTHF)_2_]^+^, whose X-ray structure is described.

## 2. Results and Discussion

### 2.1. UV-vis Absorption Spectroscopy

#### 2.1.1. Dioxygen Chemistry of [(TPP)Mn^II^]

**Figure 1:**
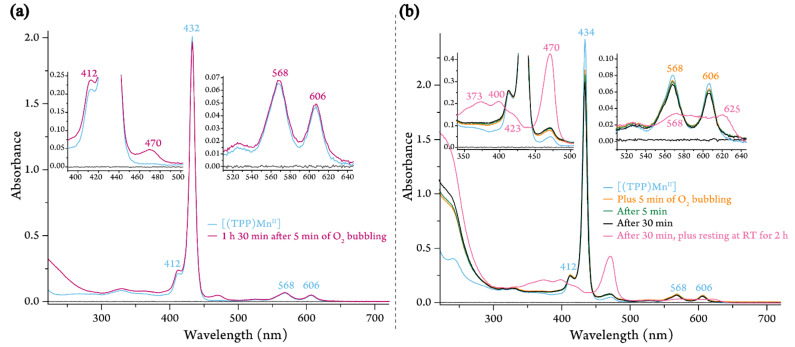
UV-vis spectra of [(TPP)Mn^II^] in MeTHF in a 4-mm cuvette upon 5 min of O_2_ bubbling at: (**a**) room temperature (10 µM), and (**b**) −90 °C (11 µM).

In coordinating solvents such as 2-methyltetrahydrofuran (MeTHF), [(TPP)Mn^II^] is rather unreactive toward O_2_ at room temperature. As shown in Figure 1a, despite long O_2_ exposure, the UV-vis spectrum of the reaction mixture mostly retained the Soret (433 nm) and Q-band (568 and 606 nm) absorption characteristics of the manganous complex. The UV-vis spectroscopic changes of [(TPP)Mn^II^] oxygenation in MeTHF at −90 °C are shown in Figure 1b. At low temperature, a small but noticeable decrease in the 434 nm Soret band, along with the formation of a minor (TPP)Mn^III^ species (*λ*_max_ = 470 nm), were observed. Allowing the solution to warm to room temperature yielded a product with features at 378, 400, 423, and 470 nm. We posit this final product as a [(TPP)Mn^III^(MeTHF)_2_]^+^ species, based on its characteristic “split Soret band” and other absorption features similar to those of an authentic MeTHF solution of [(TPP)Mn^III^(THF)_2_]SbF_6_ (*λ*_max_ = 377, 399, 418, 467 nm) (Figures S1 and S2). We note that in the high-spin manganese(III) porphyrins, the normally intense Soret band splits into two less-intense bands; one of which is a prominent peak at lower energy (~470 nm), and the other one is a very broad band that occurs at higher energy (~380 nm). The ratios of bands well as the *λ*_max_ values for these bands are particularly sensitive to the nature of the axial ligand(s) [32,33]. The X-ray structure of [(TPP)Mn^III^(MeTHF)_2_]SbF_6_, as well as ^1^H-NMR and IR data further support the identity of this product species as [(TPP)Mn^III^(MeTHF)_2_]^+^ (vide infra).

#### 2.1.2. Dioxygen Chemistry of [(TPP)Mn^II^]/[(tmpa)Cu^I^(MeCN)][B(C_6_F_5_)_4_]

**Figure 2:**
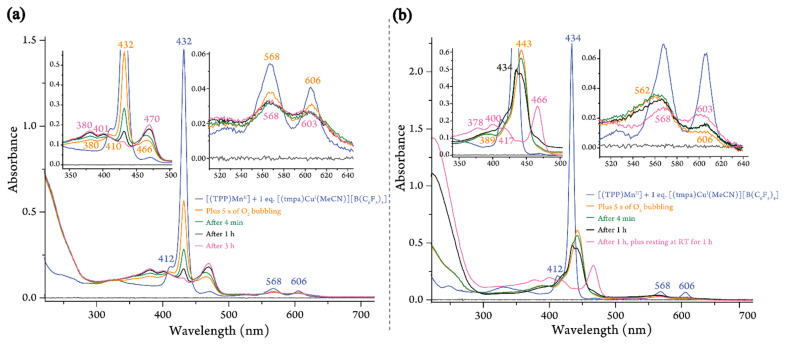
UV-vis spectra of the oxygenation reaction of 1:1 mixture of [(TPP)Mn^II^] and [(tmpa)Cu^I^(MeCN)][B(C_6_F_5_)_4_] in MeTHF in a 4-mm cuvette at: (**a**) room temperature (7 µM), and (**b**) −110 °C (8 µM).

While [(TPP)Mn^II^] showed no appreciable reactivity toward O_2_ at room temperature, remarkably, equimolar amounts of [(TPP)Mn^II^] and [(tmpa)Cu^I^(MeCN)][B(C_6_F_5_)_4_] immediately reacted with O_2_ to produce a new species (Figure 2a), now to be referred to as the *μ*-oxo Mn^III^/Cu^II^ assembly, as monitored by UV-vis spectroscopy (*λ*_max_ = 432 and 466 nm). The nature of this bridged complex was further supported by ESI-MS and ^1^H-NMR results, vide infra. This complex gradually decomposes to a final (TPP)Mn^III^ species (*λ*_max_ = 470 nm) with features similar to that of the [(TPP)Mn^II^] oxygenation product, i.e., [(TPP)Mn^III^(MeTHF)_2_]^+^.

Investigations of the independent O_2_ chemistry for the copper complex, [(tmpa)Cu^I^(MeCN)][B(C_6_F_5_)_4_], have been described previously [28,30]. The O_2_ adduct observed from low-temperature reactions of this copper complex bearing the tripodal TMPA ligand with O_2_ was solvent-, temperature-, and concentration-dependent (Cu/O_2_ = 1:1 or 2:1). Upon initial binding of O_2_ to [(tmpa)Cu^I^(MeCN)]^+^, an end-on cupric *η^1^*-superoxo intermediate, [(tmpa)Cu^II^(O_2_^•–^)]^+^, was formed via single electron transfer from the cuprous center to the ligated dioxygen moiety. At higher concentrations of the copper complex solution, and/or higher reaction temperatures, a second cuprous center provided the second electron, generating a dicopper end-on *μ-1,2*-peroxo species, [(tmpa)Cu^II^–(O_2_^2–^)–Cu^II^(tmpa)]^2+^.

With this background, we investigated the oxygenation of an equimolar mixture of [(TPP)Mn^II^] and [(tmpa)Cu^I^(MeCN)][B(C_6_F_5_)_4_] at low temperatures. At −110 °C, bubbling with dioxygen immediately produced a metastable O_2_-adduct with new spectral features at 389, 443, 562, and 606 nm (Figure 2b). We formulated this as a heterobinuclear peroxo complex, [(tmpa)Cu^II^–(O_2_^2–^)–Mn^III^(TPP)]^+^. The reaction proceeded through initial formation of a cupric superoxo species, [(tmpa)Cu^II^(O_2_^•–^)]^+^, followed by a fast electron transfer from the manganous center to the superoxide moiety, forming the bridged peroxo species. This *μ*-peroxo Mn^III^/Cu^II^ complex was not stable at higher temperatures, primarily leading to the formation of a *μ*-oxo complex, [(tmpa)Cu^II^–O–Mn^III^(TPP)]^+^. By analogy to the process shown to occur in similar bridged peroxo systems, it was presumed that this transformation occurred through a disproportionation reaction of two *μ*-peroxo species that generated two *μ*-oxo complexes and released O_2_ [27,34]. 

Warming the *μ*-peroxo complex, [(tmpa)Cu^II^–(O_2_^2–^)–Mn^III^(TPP)]^+^, to room temperature leads to the formation of the *μ*-oxo complex that subsequently converts to [(TPP)Mn^III^(MeTHF)_2_]^+^, i.e., the identical (TPP)Mn^III^ product obtained from the oxygenation experiment at room temperature. 

It is important to note that, under the given experimental conditions, i.e., low temperature (−110 °C) and low concentration (μM range), the oxygenation reaction of [(tmpa)Cu^I^(MeCN)][B(C_6_F_5_)_4_] exclusively resulted in the formation of the cupric superoxo species [29,30,35]. This strongly suggests that the electron transfer from a second cuprous complex to [(tmpa)Cu^II^(O_2_^•–^)]^+^ is significantly less favored than from a [(TPP)Mn^II^] complex. We can, therefore, rule out the formation of the dicopper peroxo species, i.e., [(tmpa)Cu^II^–(O_2_^2–^)–Cu^II^(tmpa)]^2+^, in our experiments, independent of the experimental conditions. 

#### 2.1.3. Dioxygen Chemistry of [(TPP)Mn^II^]/2 [(tmpa)Cu^I^(MeCN)][B(C_6_F_5_)_4_]

**Figure 3:**
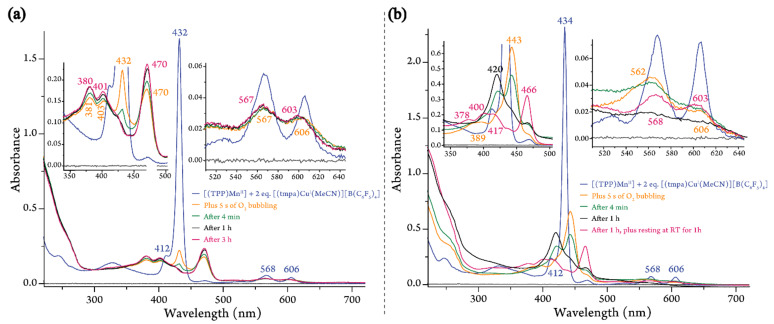
UV-vis spectra of the oxygenation reaction of 1:2 mixture of [(TPP)Mn^II^] and [(tmpa)Cu^I^(MeCN)][B(C_6_F_5_)_4_] in MeTHF in a 4-mm cuvette at: (**a**) room temperature (7 µM), and (**b**) −110 °C (8 µM).

The remaining question about the capability of the manganese(II) porphyrin precursor to react with more than an equimolar amount of the superoxide was addressed by reacting a 1:2 mixture of [(TPP)Mn^II^] and [(tmpa)Cu^I^(MeCN)][B(C_6_F_5_)_4_] with dioxygen. Here, at room temperature, the oxygenation reaction and formation of the final solvated (TPP)Mn^III^ product were faster compared with those of an equimolar mixture, with nearly all of [(TPP)Mn^III^(MeTHF)_2_]^+^ formed within 1 h after O_2_ bubbling rather than over the 3 h observed for the 1:1 mixture (Figure 2a and Figure 3a).

The same reaction was repeated at −110 °C, where we were able to detect two intermediates. Low temperature oxygenation of the 1:2 mixture leads to rapid formation of the *μ*-peroxo [(TPP)Mn^III^−(O_2_^2−^)−Cu^II^(tmpa)]^+^ intermediate (*λ*_max_ = 443 nm), identical to that observed during the reaction of the 1:1 mixture with O_2_, vide supra. This *μ*-peroxo Mn^III^/Cu^II^ intermediate then reacted with a second cupric superoxo species, [(tmpa)Cu^II^(O_2_^•–^)]^+^, on the open face of the Mn-porphyrin (Scheme 1) and isosbestically converted to a *bis*-*μ*-peroxo adduct with *λ*_max_ = 420 nm, i.e., [(tmpa)Cu^II^−(O_2_^2−^)−Mn^IV^(TPP)−(O_2_^2−^)−Cu^II^(tmpa)]^2^^+^ (Figure 3b). The characteristic single blue-shifted Soret absorption at 420 nm and other spectral features of this new species agreed with those of previously reported [(TPP)Mn^IV^(L)_2_] species (Table 1), supporting the oxidation state of manganese(IV) in this intermediate [17,36,37,38]. Further indication that the *bis*-*μ*-peroxo intermediate is a manganese(IV) complex was derived from the resonance observed for its pyrrolic protons using ^1^H-NMR spectroscopy, vide infra. Upon warming, the *bis*-*μ*-peroxo intermediate may disproportionate to form a *bis*-*μ*-oxo adduct, [(tmpa)Cu^II^−O−Mn^IV^(TPP)−O−Cu^II^(tmpa)]^2+^, which can subsequently decompose to the observed (TPP)Mn^III^ product, [(TPP)Mn^III^(MeTHF)_2_]^+^ (Figure 3b).

It is worth mentioning that quantitative analyses of UV-vis spectra of the oxygenation products of [(TPP)Mn^II^] in the presence of 0 to 2 equivalents of [(tmpa)Cu^I^(MeCN)]^+^, at either low or room temperature, confirmed the generation of one equivalent of the final product, [(TPP)Mn^III^(MeTHF)_2_]^+^, with features near 380, 400, and 470 nm in high yields (~100% yield).

### 2.2. X-ray Structure of [(TPP)Mn^III^(MeTHF)_2_]SbF_6_

**Figure 4:**
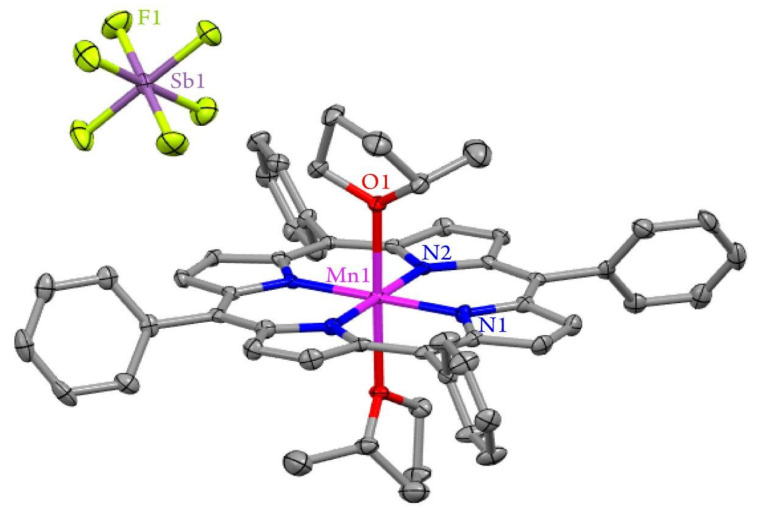
Displacement ellipsoid plot (50% probability level) of [(TPP)Mn^III^(MeTHF)_2_]SbF_6_ at 100(2) K, showing the atom-labeling scheme. Hydrogen atoms and solvent molecules have been omitted for clarity. Selected bond lengths (Å) and angles (deg): Mn(1)-O(1), 2.272(3); Mn(1)-N(1), 2.007(4); Mn(1)-N(2), 2.008(4); O(1)-Mn(1)-O(1a), 177.51(17); N(1)-Mn(1)-N(2), 90.15(17); N(1)-Mn(1)-N(1a), 89.8(2); N(1)-Mn(1)-N(2a), 179.91(19); N(1)-Mn(1)-O(1), 88.43(14); N(1)-Mn(1)-O(1a), 89.81(14); N(2)-Mn(1)-N(2a), 89.9(2); N(2)-Mn(1)-O(1), 90.21(14); N(2)-Mn(1)-O(1a), 91.55(14); N(1a)-Mn(1)-O(1), 89.80(14); N(2a)-Mn(1)-O(1), 91.55(14).

For the present studies, the molecular structure of an authentic sample of the proposed final product, [(TPP)Mn^III^(MeTHF)_2_]^+^, was also obtained. Dark red crystals of [(TPP)Mn^III^(MeTHF)_2_]SbF_6_·2MeTHF were grown by slow diffusion of heptane into a MeTHF solution of [(TPP)Mn^III^(THF)_2_]SbF_6_ (see Materials and Methods, Table S1). The complex crystallizes in a tetragonal crystal system with the *P*4_3_2_1_2 space group. A perspective view of the complex, along with the selected structural and geometrical parameters, are given in Figure 4; the molecular packing in the unit cell is shown in Figure S3. The hexacoordinate Mn center lies perfectly in the plane of the porphyrin and is axially ligated by two MeTHF molecules. The length of the bond between the manganese and the axially-ligated MeTHF molecule, Mn−O_ax,_, of 2.272(3) Å was within the range reported for other hexacoordinate Mn(III) porphyrins with two O-based ligands (Table S2) [42,43,44,45,46,47]. Moreover, the longer Mn−O_ax_ distances, as compared to Mn−N_por_ (i.e., average of 2.008 Å) were in accord with the presence of a high-spin tetragonally elongated Mn(III) center. The elongation of the bonds to the axial oxygen sites has been ascribed to a singly occupied axially antibonding *d_z_^2^* orbital that renders a ground electronic state of (*d_xz_*, *d_yz_*)^2^(*d_xy_*)^1^(*d_z2_*)^1^ [48,49,50].

### 2.3. Nuclear Magnetic Resonance (NMR) Spectroscopy

The oxygenation reactions of 1:1 and 1:2 mixtures of [(TPP)Mn^II^] and [(tmpa)Cu^I^(MeCN)]^+^ were monitored by ^1^H-NMR spectroscopy at room temperature. In both cases, NMR data support the formation of copper-bound manganese porphyrin intermediates, as well as the identity of [(TPP)Mn^III^(MeTHF)_2_]^+^ as the final product. Here, the resonances of the *β*-pyrrole protons can aid manganese oxidation and spin-state assignments. Our tentative assignments of the pyrrolic peaks are based on analogy to values reported in the literature or comparison with authentic samples (Figures S4–S10).

**Figure 5:**
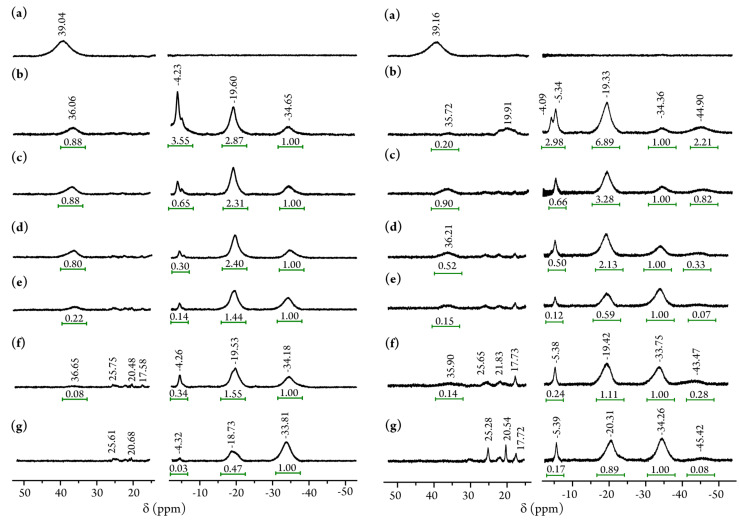
Parts of the ^1^H-NMR spectra of the oxygenation reaction of a 1:1 mixture, (**Left**), and 1:2 mixture, (**Right**), of [(TPP)Mn^II^] and [(tmpa)Cu^I^(MeCN)][B(C_6_F_5_)_4_] (THF-*d*_8_, room temperature) at different time intervals: (**a**) 0 min, (**b**) 1 min, (**c**) 15 min, (**d**) 1 h 30 min, (**e**) 4 h 30 min, (**f**) 6 h, and (**g**) 18 h. The peak for [(TPP)Mn^III^(THF)_2_]^+^ at δ = −34.65 ppm was set as the reference for comparing peak areas.

The ^1^H-NMR spectrum of an equimolar mixture of [(TPP)Mn^II^] and [(tmpa)Cu^I^(MeCN)]^+^ in THF-*d_8_* displays distinct signals originating from individual reduced complexes. The broad peak at δ = 39.04 ppm corresponds to the *β*-pyrrole protons of the Mn(II) species while the broad signal at δ = 8.02 ppm originates from the *meso*-aryl protons in [(TPP)Mn^II^] (Figure 5a Left and Figure S11a Left). The peaks at δ = 8.60, 7.74, and 7.34 ppm arise from the pyridyl groups of [(tmpa)Cu^I^(MeCN)][B(C_6_F_5_)_4_] (Figure S11a Left). The peak assignments were obtained by comparing the ^1^H-NMR spectral pattern of the reaction mixture with the individual spectra of the reduced complexes (Figures S6 and S10).

Upon O_2_ bubbling, several new signals appeared in the upfield and downfield regions in the spectrum, which resulted from oxidation of the two metal centers (Figure 5 Left and Figure S11). After 1 min of O_2_ bubbling, the ^1^H-NMR spectrum showed a distinct pyrrolic peak at δ = 36.06 ppm, which corresponds to the presence of a peroxo Mn(III) center [24,51], in agreement with our UV-vis spectroscopic results (vide supra). This proposed *μ*-peroxo Mn^III^/Cu^II^ intermediate was unstable and the corresponding pyrrolic resonance disappeared over time (Figure 5 Left). 

The upfield region displayed the three most intense peaks at δ = −4.23, −19.60, and −34.65 ppm. The signal at δ = −4.23 ppm likely originated from the TMPA moiety of the attached copper center as the significant upfield shift of this peak indicated that the protons of this chelate were directly located above the porphyrin core, as observed in similar oxo-bridged heterobinuclear systems [52,53,54]. The second upfield broad signal at δ = −19.60 ppm could be ascribed to [(TPP)Mn^III^−O−Cu^II^(tmpa)]^+^ and may correspond to a combination of the pyrrolic, as well as part of the attached, TMPA protons. The third upfield signal at δ = −34.65 ppm corresponded to the pyrrolic protons of [(TPP)Mn^III^(THF)_2_]^+^ in the reaction mixture, which was identical to that of the authentic [(TPP)Mn^III^(THF)_2_]^+^ sample, confirming the presence of a high-spin (*S* = 2) Mn(III) center (Figure S8). All proton peaks were integrated with respect to the δ = −34.65 ppm peak for relative comparisons. As the reaction progressed, the signal at δ = −34.65 ppm continued growing and the intensities of the other signals in the upfield region decreased, which suggested decomposition to [(TPP)Mn^III^(THF)_2_]^+^, consistent with the overall reactivity patterns observed in our UV-vis studies. Appearance of the small peaks in the δ = 10 to 26 ppm region over time (Figure 5 left and Figure S11b–g Left) that correspond to the TMPA protons in a free [(tmpa)Cu^II^(X)]^+^ complex [52] further confirmed the dissociation of the Mn/Cu assembly. 

To further support our supposition that an individual homobinuclear copper species did not form during the oxygenation of the mixture of manganese and copper complexes, we also monitored the independent dioxygen reactivity of [(tmpa)Cu^I^(MeCN)][B(C_6_F_5_)_4_] through ^1^H-NMR spectroscopy (Figures S12 and S13). The cuprous complex showed three distinct signals between δ = 8.84 and 7.48 ppm, corresponding to the protons of the pyridyl arms, while the methyl-protons resonated at δ = 4.53 ppm. Bubbling with O_2_ led to the formation of a dioxygen adduct with new features. The pyridyl signals appeared at δ = 11.02, 8.16, 8.11, and 7.79 ppm, while the methyl-protons resonated at δ = 5.80; this spectral pattern is in agreement with the presence of the *µ*-peroxo [(tmpa)Cu^II^–(O_2_^2–^)–Cu^II^(tmpa)]^2+^ complex in the reaction mixture [55]. The spectrum also showed an additional peak at δ = 10.39 ppm, which corresponded to the pyridyl protons of a [(tmpa)Cu^II^(X)]^+^ species [52]. Within a couple of hours, the *µ*-peroxo complex fully decomposed into the [(tmpa)Cu^II^]^+^ species, as evidenced by the gradual disappearance of the peroxo species signals and concomitant growth of the δ = 10.39 ppm peak. No proton signals in the negative region were observed throughout the oxygenation reaction of the cuprous complex; the final cupric complex only displayed proton signals in the downfield region.

The ^1^H-NMR spectral pattern obtained for a 1:2 mixture of [(TPP)Mn^II^] and [(tmpa)Cu^I^(MeCN)][B(C_6_F_5_)_4_] was quite similar to that obtained for their equimolar mixture, aside from the expected higher relative peak intensities observed for the pyridyl-protons of the cuprous complex (δ = 8.62, 7.73, and 7.35 ppm) as compared to the *meso*-aryl protons (δ = 8.02 ppm) of the porphyrin ring (Figure S11a Right). Moreover, the very broad -CH_2_- signal of (tmpa)Cu^I^ appeared at δ = 3.99 ppm. Similarly, dioxygen was bubbled through the 1:2 reaction mixture and the proton NMR spectra were recorded at different time intervals (Figure 5 Right and Figure S11b–g Right). After one minute, the sample exhibited a series of new peaks in both upfield and downfield regions. While some of the spectral features were similar to those observed for the oxygenation of the 1:1 reaction mixture, there were a few important differences. A broad peak at δ = −44.90 ppm was observed, which could be ascribed to the *β*-pyrrole protons of a high-spin (*S* = 3/2) Mn(IV) center [36,41], possibly [(tmpa)Cu^II^−(O_2_^2−^)−Mn^IV^(TPP)−(O_2_^2−^)−Cu^II^(tmpa)]^2+^ or the *bis*-*μ*-oxo adduct. This broad peak is absent during oxygenation of a 1:1 reaction mixture; this finding is in agreement with our supposition concerning the formation of a manganese(IV) species during the oxygenation reaction mixture only when an additional equivalent [(tmpa)Cu^II^(O_2_^•–^)]^+^ is present, thus, promoting the second electron transfer. This peak at δ = −44.90 slowly disappeared with a concurrent increase of the peak at δ = −34.36 ppm over the course of the reaction, hinting towards the decomposition of the Mn(IV) assembly into the (TPP)Mn^III^ species. As expected, the TMPA signals associated with a free [(tmpa)Cu^II^(X)]^+^ complex (possibly X = OH^–^) in the δ = 10 to 26 ppm region appeared at higher intensity (Figure 5 Right and Figure S11b–g Right), further supporting the dissociation of the Mn/Cu assembly.

### 2.4. Electrospray Ionization Mass Spectrometry (ESI-MS)

**Figure 6:**
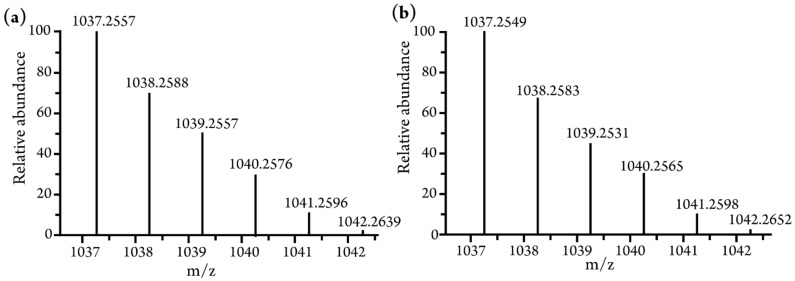
Isotopic distribution pattern of the (**a**) experimental, and (**b**) simulated ESI-MS spectra of [(TPP)Mn–O–Cu(tmpa)+H]^+^. The sample was prepared in MeTHF and recorded in positive-ion mode with a source voltage of 1.0 kV.

The formation of the *μ*-oxo [(TPP)Mn^III^−O−Cu^II^(tmpa)]^+^ complex was further confirmed by ESI-MS studies. The ESI-MS spectrum of a 1:1 mixture of [(TPP)Mn^II^] and [(MeCN)Cu^I^(tmpa)][B(C_6_F_5_)_4_] in MeTHF immediately after bubbling with O_2_ at room temperature showed a peak for [(TPP)Mn^II^−OH−Cu^II^(tmpa)]^+^ at *m*/*z* 1037.2557. The isotopic distribution pattern of the experimental mass correlated well with theory (Figure 6), which further confirms the formation of the Mn/Cu assembly. To try to provide further insight, the oxygenation of a 1:2 mixture of the reduced complexes did not lead to the mass spectrometric detection of a *bis*-*μ*-oxo adduct, most likely due to its even lower stability and, therefore, easier reduction/fragmentation.

### 2.5. Fourier-Transform Infrared Spectroscopy (FT-IR)

IR spectroscopy also supports the formation of the same final oxygenation product, i.e., [(TPP)Mn^III^(MeTHF)_2_]^+^, for 1:1, as well as 1:2, mixtures of [(TPP)Mn^II^] and [(tmpa)Cu^I^(MeCN)][B(C_6_F_5_)_4_] (Figures S14–S17). The presence of bound MeTHF in [(TPP)Mn^III^(MeTHF)_2_]^+^ was confirmed by C-H stretching bands at or near 2966 and 2864 cm^−1^. The high-frequency metal-sensitive IR bands of the TPP complexes have been previously found to, slightly but regularly, vary depending on the nature of the metal ion [56]. Here, the metal-sensitive TPP bands at 1598, 1487, 1342, and 1010 cm^−1^ were identical or within 2 cm^−1^ for the 1:1 and 1:2 oxygenation products, and similar to those of authentic [(TPP)Mn^III^(MeTHF)_2_]SbF_6_ (Table S3), further confirming the presence of a Mn(III) center.

## 3. Conclusions

The foregoing results demonstrate the cooperative activation of dioxygen by the copper(I)/manganese(II)-porphyrin systems. A combined array of variable-temperature UV-vis, NMR, IR as well as ESI-MS and X-ray crystallographic analyses collectively demonstrated that [(TPP)Mn^II^] reacts with O_2_ in the presence of 0 to 2 equivalents of a cuprous chelate, [(tmpa)Cu^I^(MeCN)]^+^, in MeTHF to produce a common final decomposition product, [(TPP)Mn^III^(MeTHF)_2_]^+^. However, the O_2_ reactivity in the presence of the copper(I) center is remarkably faster and proceeds through a distinct mechanism. As evidenced by low temperature (−110 °C) UV-vis and room temperature ^1^H-NMR measurements, copper(I) is responsible for the binding and initial activation of dioxygen, forming a cupric superoxo intermediate, [(tmpa)Cu^II^(O_2_^•–^)]^+^. This is followed by an electron transfer from the manganous complex generating a bridged heterobinuclear peroxo species, [(tmpa)Cu^II^–(O_2_^2–^)–Mn^III^(TPP)]^+^. In the presence of a second equivalent of the cupric superoxo complex, an additional electron transfer from the Mn(III) center of the *μ*-peroxo assembly to the superoxo species takes place, forming a *bis*-*μ*-peroxo assembly, [(tmpa)Cu^II^−(O_2_^2−^)−Mn^IV^(TPP)−(O_2_^2−^)−Cu^II^(tmpa)]^2+^. These systems represent the first examples of such Mn/Cu/O_2_ adducts and illustrate the importance of cooperative activation of O_2_ in the heteromultimetallic systems. Experiments designed to further investigate these dioxygen-adducts are currently in hand.

## 4. Materials and Methods

### 4.1. General Methods

Chemicals were purchased commercially and used without further purification, unless noted otherwise. Acetonitrile (MeCN), dichloromethane (DCM), tetrahydrofuran (THF), and 2-methyltetrahydrofuran (MeTHF) were purchased from Sigma-Aldrich (St. Louis, MO, USA). Deuterated solvents (acetone-*d_6_*, CD_2_Cl_2_, and THF-*d_8_*) were purchased from Cambridge Isotope Laboratories (Tewksbury, MA, USA). Commercial ACS grade solvents were used for chromatography and extractions. For the reactions, all solvents were purified by an Innovative Technologies (Newburyport, MA, USA) or Inert PureSolv Micro (Amesbury, MA, USA) solvent purification system. Solvents were then deoxygenated by bubbling with argon for 1 h, followed by storage over 3 or 5 Å molecular sieves for at least 72 h prior to use. Deionized water was purified by a PURELAB flex 1 Analytical Ultrapure Water System (ELGA) to obtain nanopure water with a specific resistance of 18.2 MΩ cm at room temperature. Air-sensitive compounds were prepared and handled under a dry, oxygen-free argon atmosphere using standard Schlenk techniques or under nitrogen atmosphere in a Vacuum Atmospheres (Hawthorne, CA, USA) OMNI-Lab inert atmosphere (<0.5 ppm of O_2_ and H_2_O) glovebox. Ultra-high purity grade oxygen gas was purchased from Airgas (Greensboro, NC, USA) and dried by passing through a drying column containing Drierite desiccant and 3 Å activated molecular sieves. For the NMR experiments, dry O_2_ gas was transferred and stored in a capped 50-mL Schlenk flask, then added into the metal complex solutions via a three-way long syringe needle. 

UV-vis absorption spectra were recorded on an Agilent (Wilmington, DE, USA) Cary-60 spectrophotometer equipped with a Unisoku (Osaka, Japan) CoolSpeK USP-203-B cryostat using 4-mm modified Schlenk cuvettes. Infrared (IR) spectra of neat solid samples were obtained using a Thermo Scientific (West Palm Beach, FL, USA) Nicolet iS5 Fourier Transform IR (FT-IR) spectrometer equipped with an iD7 attenuated total reflection (ATR) accessory. NMR spectra were recorded on a JEOL (Peabody, MA, USA) 500 MHz spectrometer, and the chemical shifts were referenced against NMR solvent residual shifts (e.g., THF-*d_8_* at 𝛿 = 1.72 ppm) and/or tetramethylsilane (TMS at 𝛿 = 0.00 ppm). Electrospray ionization mass spectrometry (ESI-MS) data was collected in positive ion mode on a Thermo Fisher Scientific (Waltham, MA, USA) Q Exactive Plus system.

### 4.2. Synthesis and Characterization

The compounds [(tmpa)Cu^I^(MeCN)][B(C_6_F_5_)_4_] [57] and [(TPP)Mn^III^Cl] [58] were synthesized and characterized following previously described methods. 

#### 4.2.1. Synthesis of [(TPP)Mn^II^]

The complex was prepared following slight modification of the literature procedure reported for the ferric choro complexes bearing similar porphyrin rings [59,60]. Using standard Schlenk techniques, a solution of [(TPP)Mn^III^Cl] (500 mg, 0.711 mmol) in DCM (200 mL) was mixed with a solution of sodium dithionite (22 g, 0.126 mol) in water (100 mL) for 1 h by bubbling argon. The reaction mixture was allowed to sit for approximately 20 min to allow the separation of the two layers. The DCM layer was filtered through sodium sulfate to remove residual water, then dried under vacuum, producing the deep-purple microcrystalline product. Yield: 416 mg, 91%. UV-vis (*λ*_max_, nm [ε, M^−1^cm^−1^]): 327 (22,800), 412 (54,300), 432 (495,000), 527 (4000), 568 (16,600), 606 (11,300) in MeTHF; 327 (f,000), 412 (53,800), 432 (492,000), 527 (3000), 568 (15,500), 606 (11,300) in THF. ^1^H-NMR (THF-*d_8_*, δ, ppm): 38.67 (s, 8H, *β*-pyrrole), 8.01 and 7.84 (s, 20H, phenyl).

#### 4.2.2. Synthesis of [(TPP)Mn^III^(THF)_2_]SbF_6_

The complex was prepared following slight modification of the reported procedure for a similar manganese complex [50]. In the glovebox, a solution of [(TPP)Mn^III^Cl] (101 mg, 0.138 mmol) and AgSbF_6_ (52 mg, 0.152 mmol) in THF (20 mL) was stirred for 1.5 h under reduced light at room temperature, then filtered to remove precipitated AgCl. The filtrate was concentrated under vacuum and layered with hexanes to obtain the dark-red semi-crystalline product, which was further vacuum dried. Yield: 119 mg, 83%. UV-vis (*λ*_max_, nm [ε, M^−1^cm^−1^]): 382 (52,400), 402 (46,800), 420 (31,300), 472 (67,800), 516 (6500), 565 (9800), 600 (8000) in MeTHF; 382 (51,500), 403 (46,700), 420 (31,400), 472 (64,900), 516 (6200), 565 (9700), 600 (7700) in THF. A UV-vis spectrum of [(TPP)Mn^III^(THF)_2_]SbF_6_ was also recorded in MeTHF at −110 °C for comparison (Figure S2): 377, 399, 418, 467, 514, 565, and 601 nm. ^1^H-NMR (THF-*d_8_*, δ, ppm): −34.35 (s, 8H, *β*-pyrrole), 9.65 (s, 8H, phenyl-*o*), 8.01 (s, 4H, phenyl-*p*), 7.43 (s, 8H, phenyl-*m*), 3.60 (s, 8H), 1.76 (s, 8H). IR (solid, cm^−1^): *ν*_(C-H, MeTHF)_ = 2950, 2869; *ν*_(SbF6)_ = 651.

### 4.3. Dioxygen Reactivity Studies

#### 4.3.1. Room-Temperature UV-vis Measurements for [(TPP)Mn^II^] 

Inside the glovebox, 4.8 mg (0.007 mmol) of [(TPP)Mn^II^] was dissolved in MeTHF (5 mL). Samples for UV-vis spectral measurements were prepared by diluting 7 μL of this stock solution with MeTHF to 1 mL, which was then transferred to a 4-mm modified Schlenk cuvette equipped with a septum. The spectra were recorded upon bubbling of dry O_2_ gas directly into the solution at room temperature (Figure 1a).

#### 4.3.2. Low-Temperature UV-vis Measurements for [(TPP)Mn^II^] 

A stock solution of [(TPP)Mn^II^] (5.3 mg, 0.008 mmol) in MeTHF (5 mL) was prepared inside the glovebox. UV-vis samples for low-temperature measurements were prepared by diluting 7 μL of this stock solution with MeTHF to 1 mL, which was then transferred to a 4-mm modified Schlenk cuvette equipped with a septum. The spectra were recorded upon bubbling of dry O_2_ gas directly into the solution at −90 °C (Figure 1b). The solution was monitored at −90 °C for 30 min after O_2_ bubbling, then allowed to warm up to room temperature. The solution was further monitored for 2 h, with spectra recorded at −90 °C.

#### 4.3.3. Room-Temperature UV-vis Measurements for 1:1 and 1:2 Mixtures of [(TPP)Mn^II^] and [(tmpa)Cu^I^(MeCN)][B(C_6_F_5_)_4_]

For the 1:1 mixture, 4.7 mg (0.007 mmol) of [(TPP)Mn^II^] and 7.5 mg (0.007 mmol) of [(tmpa)Cu^I^(MeCN)][B(C_6_F_5_)_4_] were dissolved in MeTHF (5 mL) inside the glovebox. Samples for UV-vis spectral measurements were prepared by diluting 5 μL of this stock solution with MeTHF to 1 mL. The UV-vis sample was then transferred to a 4-mm modified Schlenk cuvette equipped with a septum. The spectra were recorded upon bubbling of dry O_2_ gas directly into the solution at room temperature (Figure 2a). The reaction was repeated for the 1:2 mixture (Figure 3a). Here, the stock solution was prepared by dissolving 4.7 mg (0.007 mmol) of [(TPP)Mn^II^] and 15.1 mg (0.014 mmol) of [(tmpa)Cu^I^(MeCN)][B(C_6_F_5_)_4_] in MeTHF (5 mL) inside the glovebox. All other steps were identical to those described for the 1:1 mixture.

#### 4.3.4. Low-Temperature UV-vis Measurements for 1:1 and 1:2 Mixtures of [(TPP)Mn^II^] and [(tmpa)Cu^I^(MeCN)][B(C_6_F_5_)_4_] 

For the 1:1 mixture, 5.3 mg (0.008 mmol) of [(TPP)Mn^II^] and 8.5 mg (0.008 mmol) of [(tmpa)Cu^I^(MeCN)][B(C_6_F_5_)_4_] were dissolved in MeTHF (5 mL) inside the glovebox. UV-vis samples were prepared by diluting 5 μL of this stock solution with MeTHF to 1 mL. This solution was then transferred to a 4-mm modified Schlenk cuvette equipped with a septum. The spectra were recorded upon bubbling of dry O_2_ gas directly into the solution at −110 °C (Figure 2b). The solution was monitored at −110 °C for 1 h after O_2_ bubbling, then allowed to warm up to room temperature over 1 h. The spectrum of the warmed solution was recorded at −110 °C. For the 1:2 mixture, 5.3 mg (0.008 mmol) of [(TPP)Mn^II^] and 17.1 mg (0.016 mmol) of [(tmpa)Cu^I^(MeCN)][B(C_6_F_5_)_4_] were dissolved in MeTHF (5 mL) inside the glovebox. All other steps, as described for the 1:1 mixture, were repeated for this sample (Figure 3b).

#### 4.3.5. ^1^H-Nuclear Magnetic Resonance (^1^H-NMR) Measurements

To prepare the 1:1 mixture NMR sample, 7.5 mg (0.011 mmol) of [(TPP)Mn^II^] and 12.1 mg (0.011 mmol) of [(tmpa)Cu^I^(MeCN)][B(C_6_F_5_)_4_] were dissolved in THF-*d_8_* (800 μL) and transferred to an NMR tube inside the glovebox. The NMR sample sealed with a rubber septum was taken out, and the spectrum prior to dioxygen bubbling was recorded at room temperature. For oxygenation, 4 mL of dry O_2_ gas was bubbled into the metal complex solution in the NMR tube using a Hamilton gastight syringe equipped with a three-way valve. After O_2_ bubbling, the first spectrum was recorded within 2 min of mixing, and the reaction was monitored over 18 h (Figure 5 and Figure S11). The same steps were repeated for the 1:2 mixture NMR sample which was prepared by dissolving 7.5 mg (0.011 mmol) of [(TPP)Mn^II^] and 24.2 mg (0.022 mmol) of [(tmpa)Cu^I^(MeCN)][B(C_6_F_5_)_4_] in THF-*d_8_* (800 μL). 

In a control experiment, in order to confirm the O_2_ reactivity of the authentic mononuclear Cu(I) complex in our experimental conditions, NMR studies were carried out following a slightly modified version of the literature procedure [55]. Inside the glovebox, [(tmpa)Cu^I^(MeCN)][B(C_6_F_5_)_4_] (24.2 mg, 0.022 mmol) was dissolved in THF-*d_8_* (800 μL) and transferred to an NMR tube. Oxygenation of the Cu(I) complex solution was carried out using methods described above. After O_2_ bubbling, the first spectrum was recorded within 2 min of mixing; the reaction was monitored over 6 h (Figures S12 and S13). 

### 4.4. Crystallographic Studies

Suitable X-ray quality single crystals of [(TPP)Mn^III^(MeTHF)_2_]SbF_6_ were obtained by transferring a 7 µM solution of [(TPP)Mn^III^(THF)_2_]SbF_6_ in MeTHF into a 5-mm glass tube, layering with heptane, and storing it in the glovebox at room temperature. All reflection intensities were measured at 100(2) K using a Gemini R diffractometer (equipped with Atlas detector) with MoKα radiation (λ = 0.71073 Å) under the program CrysAlisPro (Version CrysAlisPro 1.171.38.43f, Rigaku OD, 2015). The same program (but a different version viz. CrysAlisPro 1.171.40.53, Rigaku OD, 2019) was used to refine the cell dimensions and for data reduction. The temperature of the data collection was controlled using the system Cryojet (manufactured by Oxford Instruments, Abingdon, UK). The structure was solved with the program SHELXT-2018/2 and was refined on F^2^ by full-matrix least-squares technique using the SHELXL-2018/3 program package [61]. Numerical absorption correction based on Gaussian integration was applied using a multifaceted crystal model by CrysAlisPro. Non-hydrogen atoms were refined anisotropically. In the refinement, hydrogen atoms were treated as riding atoms using SHELXL default parameters.

CSD 2128108 contains the supplementary crystallographic data for this paper. These data can be obtained free of charge via https://www.ccdc.cam.ac.uk/data_request/cif.

### 4.5. Electrospray Ionization Mass Spectrometry (ESI-MS) Measurements 

The 1:1 mixture stock solution was prepared by dissolving 7.5 mg (0.011 mmol) of [(TPP)Mn^II^] and 12.1 mg (0.011 mmol) of [(tmpa)Cu^I^(MeCN)][B(C_6_F_5_)_4_] in MeTHF (800 μL) inside the glovebox. The stock solution was brought out in a Schlenk flask equipped with a rubber septum, and dry O_2_ gas was bubbled into the mixture for 30 s. Samples for ESI-MS measurement were taken from this Schlenk flask and diluted by 20 folds in MeTHF before injection. The same procedure was repeated for the 1:2 mixture, whose stock solution was prepared by dissolving 7.5 mg (0.011 mmol) of [(TPP)Mn^II^] and 24.2 mg (0.022 mmol) of [(tmpa)Cu^I^(MeCN)][B(C_6_F_5_)_4_] in MeTHF (800 μL), followed by bubbling dry O_2_. In order to detect multinuclear assemblies, soft ionization was maintained at a relatively low source voltage of 1.0 kV.

## Figures and Tables

**Table 1:** Examples of UV-vis absorption features reported for Mn^IV^ complexes bearing TPP-based ligands.

Complex *	Solvent	λ_max_ (nm)	Reference
{(TPP)Mn^IV^[(O_2_^2−^)Cu^II^(tmpa)]_2_}^2+^ (**7**)	MeTHF	420	This work
[(TPP)Mn^IV^(OCH_3_)_2_]	methanol	360, 420, 520	[39]
[Cl(TPP)Mn^IV^(OIPh)]_2_O	chlorobenzene	421, 502	[40]
[(TMP)Mn^IV^(O)(OH)]	DCM	422	[8]
[(TMP)Mn^IV^Cl_2_]	DCM	420, 530, 620, 720	[41]
	benzene	420, 535, 720	

* TPP: Tetraphenylporphyrin; TMPA: Tris(2-pyridylmethyl)amine); TMP: Tetramesitylporphyrin.

## Data Availability

Raw data are available upon request.

## Supplementary Materials

The following supporting information can be downloaded. Details concerning spectroscopy including UV-vis, ^1^H-NMR, and IR as well as crystallographic data.

## Sample Availability

Samples of the manganese and/or copper complexes, in limited quantities, are available from the authors.
