# Supplementary material for: Dioxygen Reactivity of Copper(I)/Manganese(II)-Porphyrin Assemblies: Mechanistic Studies and Cooperative Activation of O2
Source: Molecules. 2022 Feb 1;27(3):1000. doi: 10.3390/molecules27031000 (PMC8839022; doi:10.3390/molecules27031000)
Supplement: Supplementary file 1 [file molecules-27-01000-s001.zip › molecules-1550782-supplementary/molecules-1550782-supplementary.pdf]

Supporting Information:

Dioxygen Reactivity of Copper(I)/Manganese(II)-Porphyrin Assemblies: Mechanistic Studies and Cooperative Activation of O_2

Runzi Li, Firoz Shah Tuglak Khan and Shabnam Hematian *

Department of Chemistry and Biochemistry, University of North Carolina at Greensboro, Greensboro, North Carolina 27412

1. UV-vis Spectroscopy

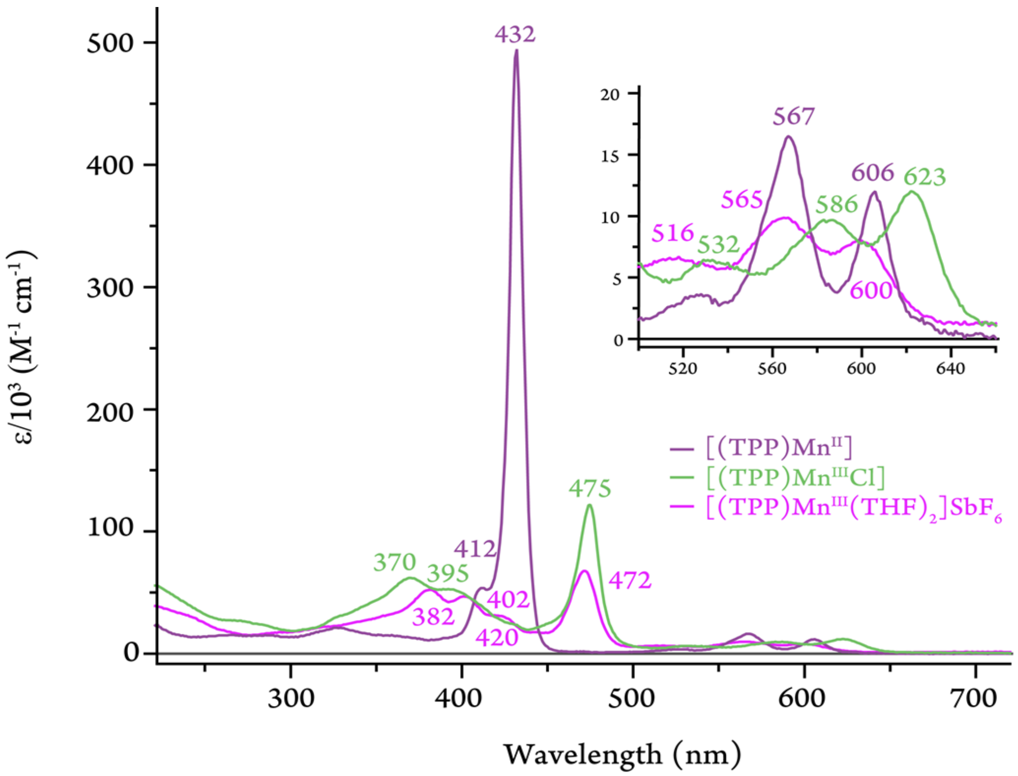

Figure S1. Quantitative electronic spectra of [(TPP)Mn^{II}] (purple), [(TPP)Mn^{III}Cl] (green), and [(TPP)Mn^{III}(THF)$_2$]SbF_6 (magenta) in MeTHF at room temperature (RT).

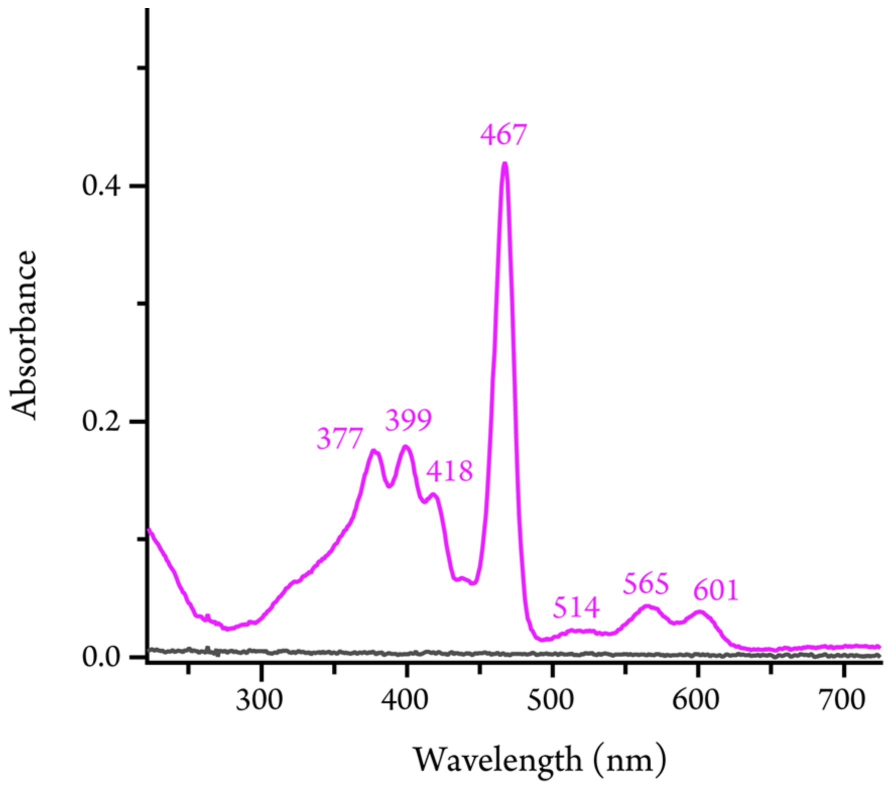

Figure S2. UV-vis spectrum of [(TPP)$Mn^{III}(THF)_2$]SbF_6 in MeTHF at –110 °C.

2. Crystallographic Studies

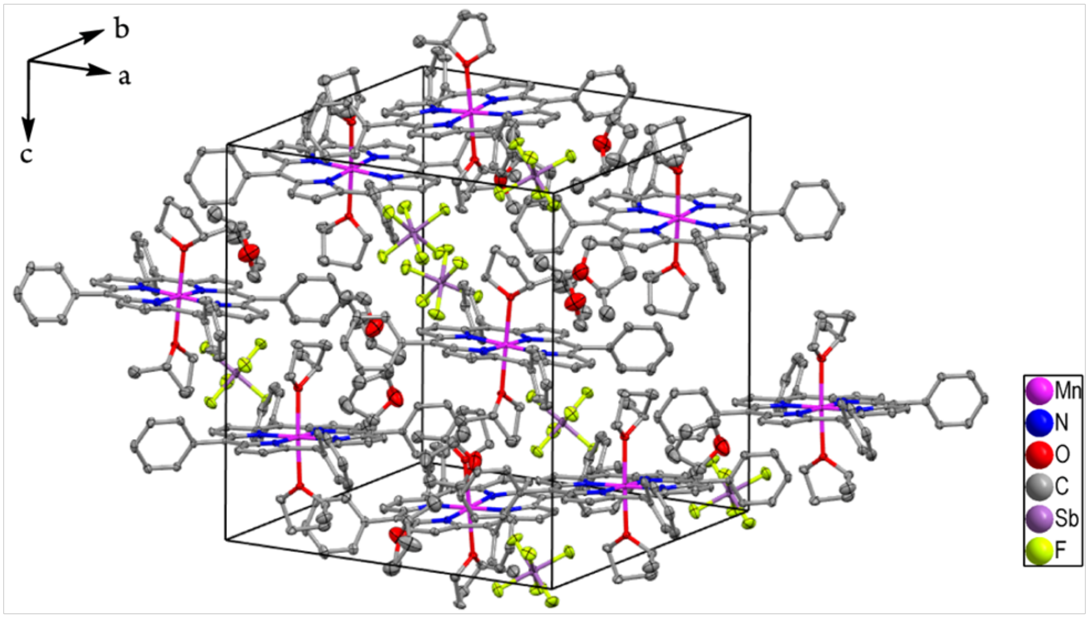

Figure S3. Diagram illustrating the molecular packing of [(TPP)$Mn^{III}(MeTHF)_2$]$SbF_6\cdot 2$MeTHF at 100(2) K (hydrogen atoms have been omitted for clarity).

Table S1. Crystallographic Data and Data Collection Parameters for $[(TPP)Mn^{III}(MeTHF)_2]SbF_6 \cdot 2MeTHF$.

Formula	$C_{64}H_{68}F_6MnN_4O_4Sb$
T (K)	100(2)
Formula weight	1247.91
Crystal system	Tetragonal
Space group	$P\ 4_3 2_1 2$
a, Å	17.4544(3)
b, Å	17.4544(3)
c, Å	18.6564(7)
α, deg	90
β, deg	90
γ, deg	90
V, $Å^3$	5683.8(3)
Z	4
Radiation (λ, Å)	MoKα (0.71073)
d_{calcd}, g•cm^{-3}	1.458
F(000)	2568
Crystal size (mm^3)	0.17 x 0.13 x 0.08
Theta range for data collection	2.334 to 25.500 °
μ, mm^{-1}	0.770
No of unique data	5299
Completeness to theta	99.9%
No. of restraints	0
No. of params. refined	377
GOF on F^2	1.015
$R1^{a\ [I>2\sigma(I)]}$	0.0359
$R1^a$ (all data)	0.0494
$wR2^b$ (all data)	0.0859
Largest diff. peak and hole	0.736 and -0.325 e.$Å^{-3}$

$$^{a}R1 = \frac{\Sigma||F_o|-|F_c||}{\Sigma|F_o|};\ ^{b}wR2 = \sqrt{\frac{\Sigma\left[w\left(F_o^2-F_c^2\right)^2\right]}{\Sigma\left[w\left(F_o^2\right)^2\right]}}$$

Table S2. Selected structural parameters for six coordinated Mn(III) porphyrin complexes having bis-axial oxygen ligation in charge neutral ligands.

Complex	Mn−O_{ax} (Å)[a]	Mn−N_{por} (Å)[a]	Reference
$[(TPP)Mn^{III}(MeTHF)_2]SbF_6$	2.272(3)	2.008(2)	This work
$[(TPP)Mn^{III}(CH_3OH)_2]ClO_4$	2.261(2)	2.006(2)	[1]
[(TPP)Mn^{III}(*N*,*N*-dimethylformamide)$_2$]ClO_4	2.217(4)	2.010(5)	[2]
[(TPP)Mn^{III}(2,6-lutidine *N*-oxide)$_2$]ClO_4	2.264(4)	1.996(4)	[3]
$[(TPP)Mn^{III}(H_2O)_2]ClO_4$	2.271(2)	2.004(2)	[4]
$[(TPP)Mn^{III}(CH_3OH)_2]SbCl_6$	2.283(5)	2.002(2)	[5]
$[(TPP)Mn^{III}(ONC_6H_4NEt_2)_2]SbF_6$	2.211(4)	2.016 (4)	[6]
[(DHPP[b])Mn^{III}(THF)$_2$]Cl	2.320(2)	2.004(2)	[7]
[(TPP)Mn^{III}(THF)$_2$][(Pc[c])Co^{III}(L[d])$_2$]	2.307(2)	2.007(2)	[8]

[a] Average value; [b] DHPP corresponds to 5,10,15,20-Tetrakis(3′,5′-dihydroxyphenyl)porphyrinato; [c] Pc corresponds to phthalocyaninato; [d] L corresponds to 1-phenyl-1H-tetrazole-5-thiolate.

3. NMR Spectroscopy

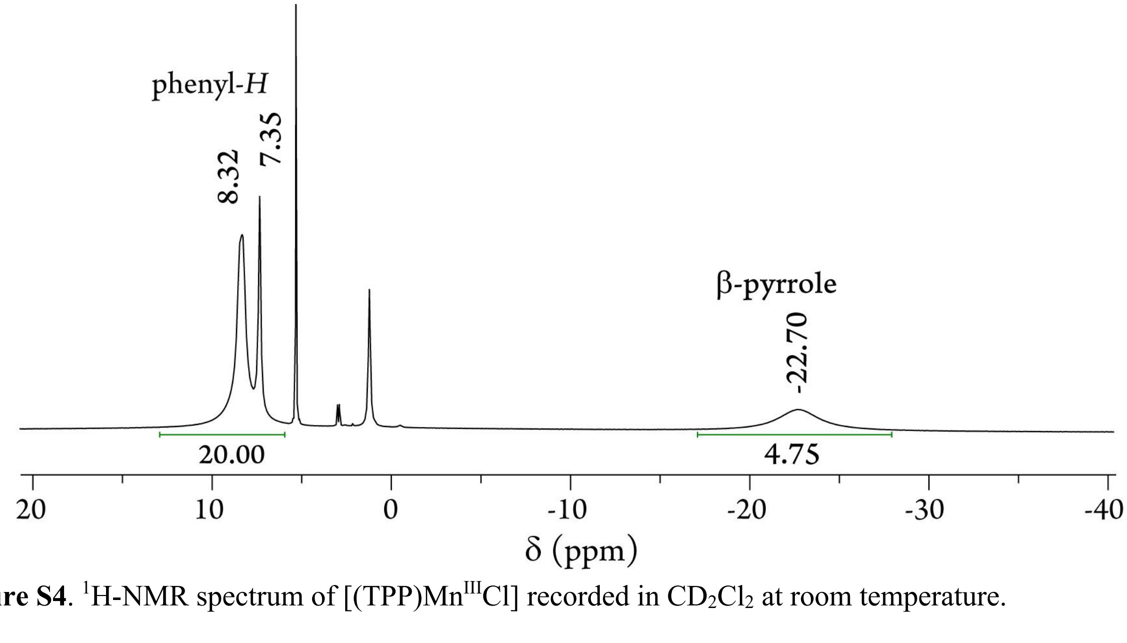

Figure S4. ^{1}H-NMR spectrum of [(TPP)Mn^{III}Cl] recorded in CD_2Cl_2 at room temperature.

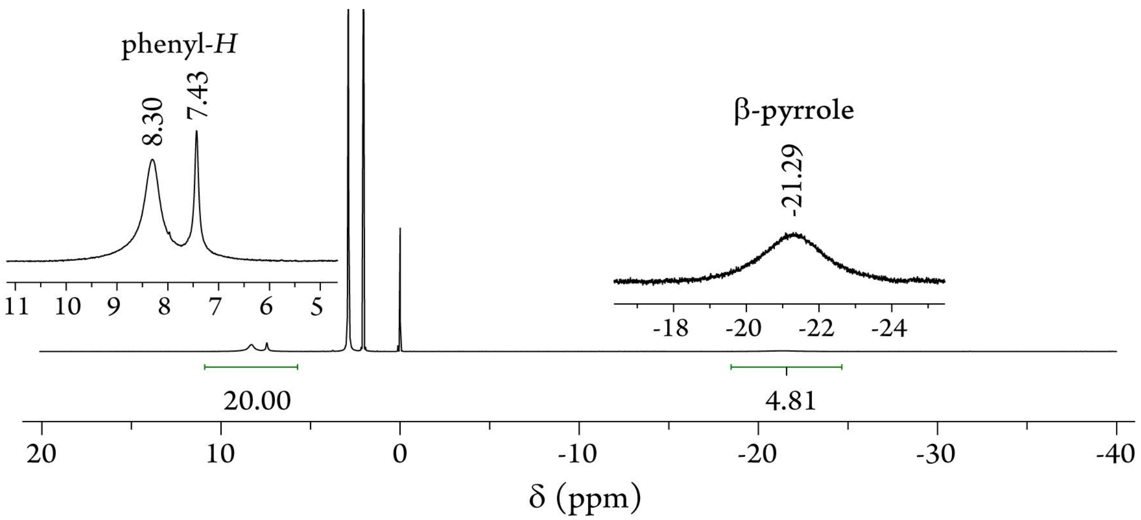

Figure S5. ^{1}H-NMR spectrum of [(TPP)Mn^{III}Cl] recorded in acetone-d_6 at room temperature.

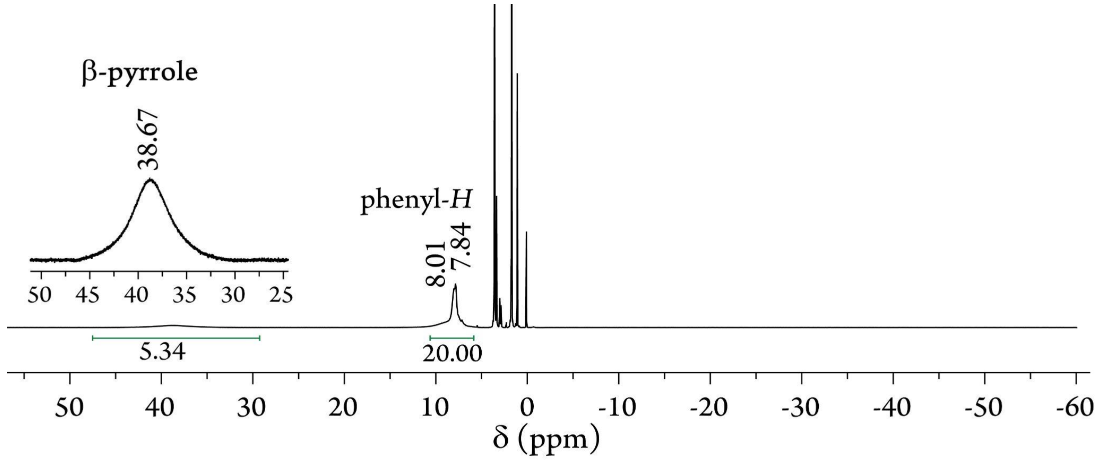

Figure S6. ^{1}H-NMR spectrum of [(TPP)Mn^{II}] recorded in THF-d_8 at room temperature.

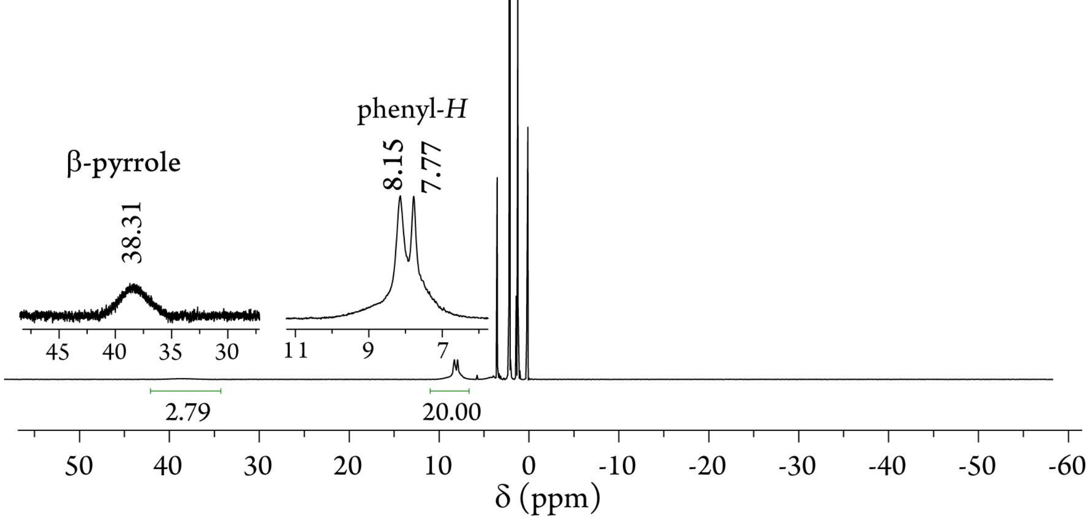

Figure S7. ^{1}H-NMR spectrum of [(TPP)Mn^{II}] recorded in acetone-d_6 at room temperature.

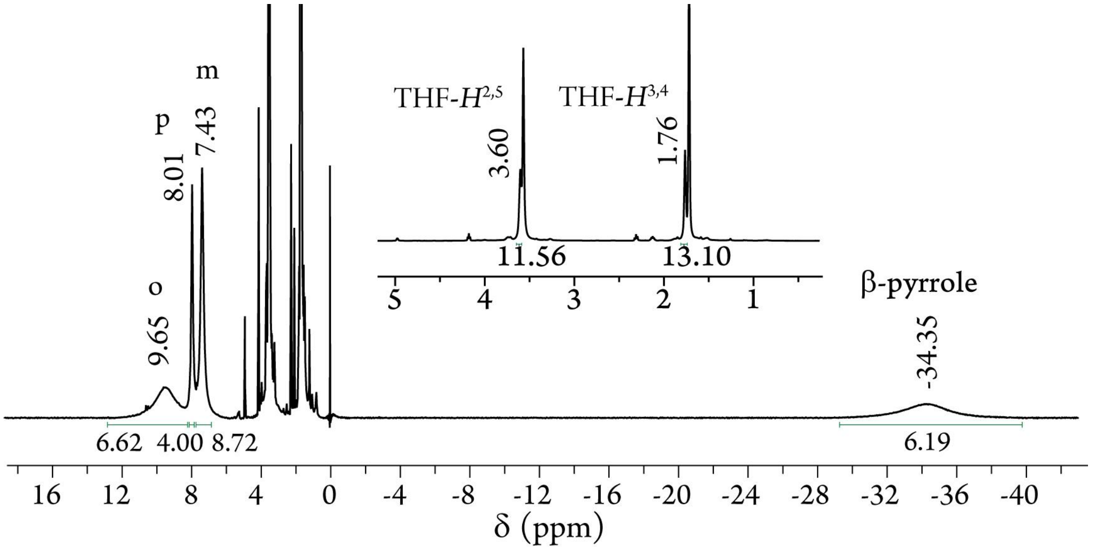

Figure S8. ^{1}H-NMR spectrum of $[(TPP)Mn^{III}(THF)_2]SbF_6$ recorded in THF-d_8 at room temperature.

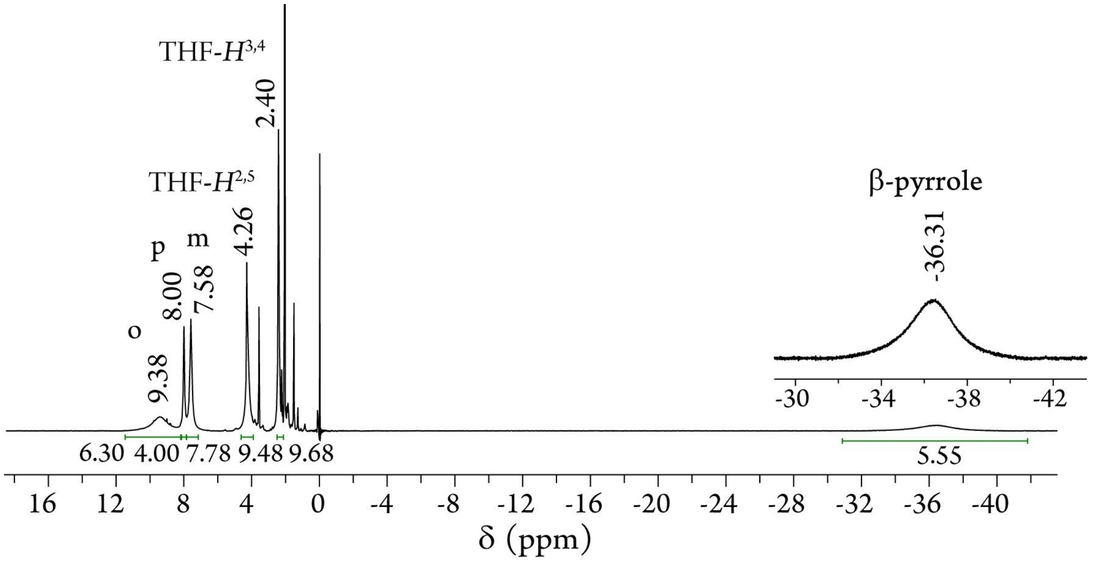

Figure S9. ^{1}H-NMR spectrum of $[(TPP)Mn^{III}(THF)_2]SbF_6$ recorded in acetone-d_6 at room temperature.

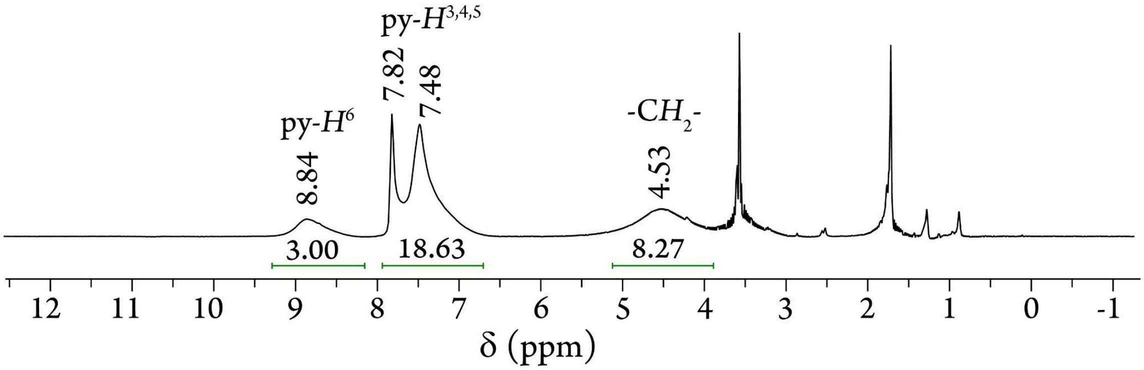

Figure S10. ^{1}H-NMR spectrum of $[(tmpa)Cu^{I}(MeCN)][B(C_6F_5)_4]$ recorded in THF-d_8 at room temperature.

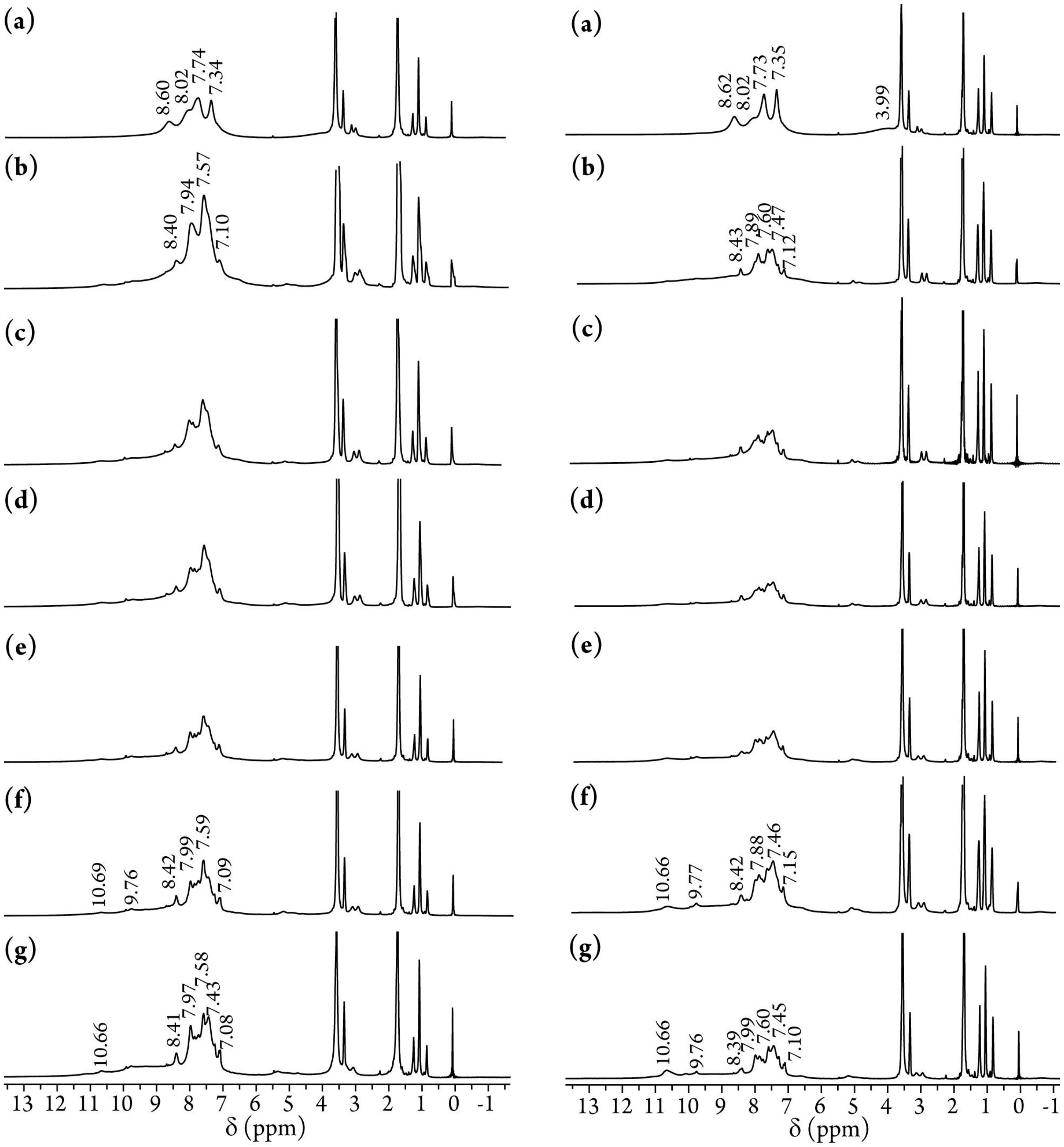

Figure S11. ^{1}H-NMR spectra (selected portions only) (THF-d_8, room temperature) of the oxygenation reaction of a 1:1 mixture, *Left*, and 1:2 mixture, *Right*, of [(TPP)Mn^{II}] and [(tmpa)Cu^I(MeCN)][B(C_6F_5)$_4$] at different time intervals: (a) 0 min, (b) 1 min, (c) 15 min, (d) 1 h 30 min, (e) 4 h 30 min, (f) 6 h, and (g) 18 h.

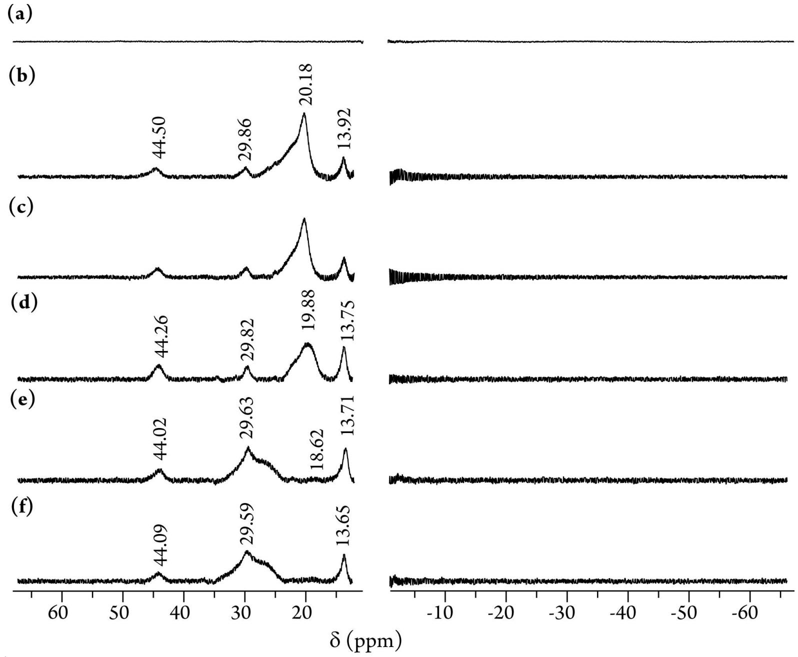

Figure S12. ^{1}H-NMR spectra (selected portions only) (THF-d_8, room temperature) of the oxygenation reaction of [(tmpa)Cu^{I}(MeCN)][B(C_6F_5)$_4$] at (a) 0 min, (b) 1 min, (c) 15 min, (d) 1h 30 min, (e) 4 h 30 min, and (f) 6 h.

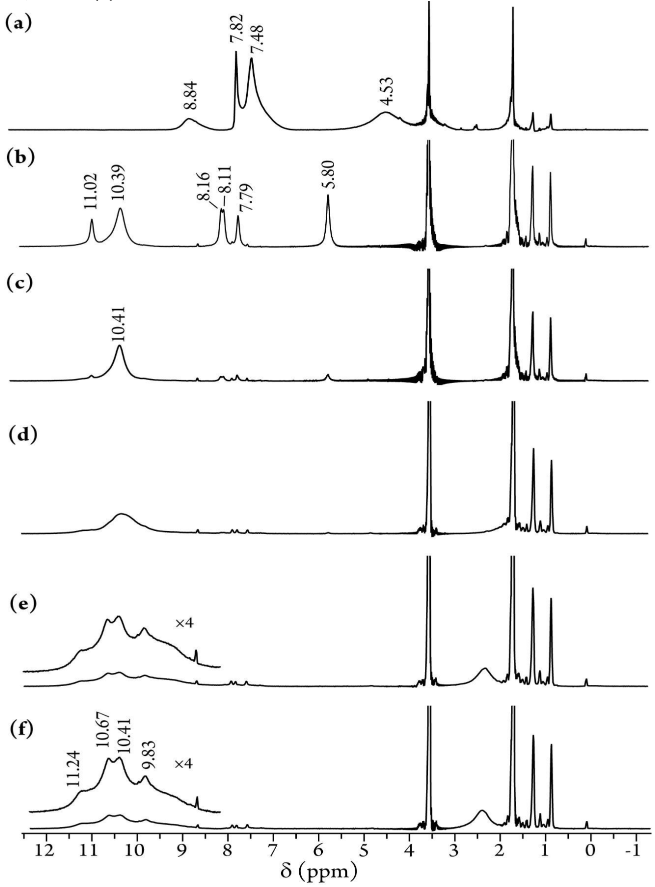

Figure S13. ^{1}H-NMR spectra (selected portions only) (THF-d_8, room temperature) of the oxygenation reaction of [(tmpa)Cu^{I}(MeCN)][B(C_6F_5)$_4$] at (a) 0 min, (b) 1 min, (c) 15 min, (d) 1 h 30 min, (e) 4 h 30 min, and (f) 6 h.

4. Infrared (IR) Spectroscopy

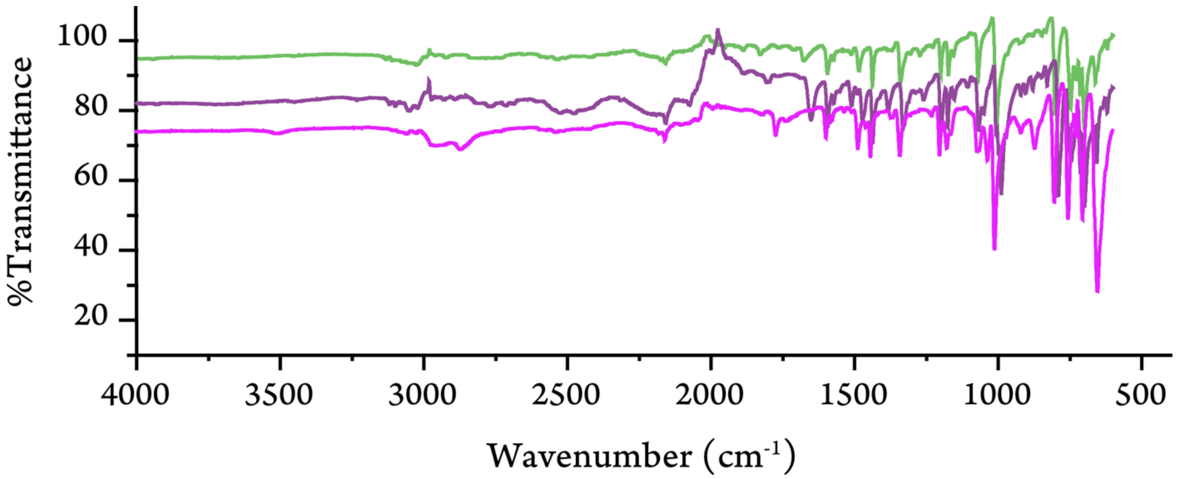

Figure S14. IR spectra of [(TPP)Mn^{II}] (purple), [(TPP)Mn^{III}Cl] (green), and [(TPP)$Mn^{III}(THF)_2$]SbF_6 (magenta).

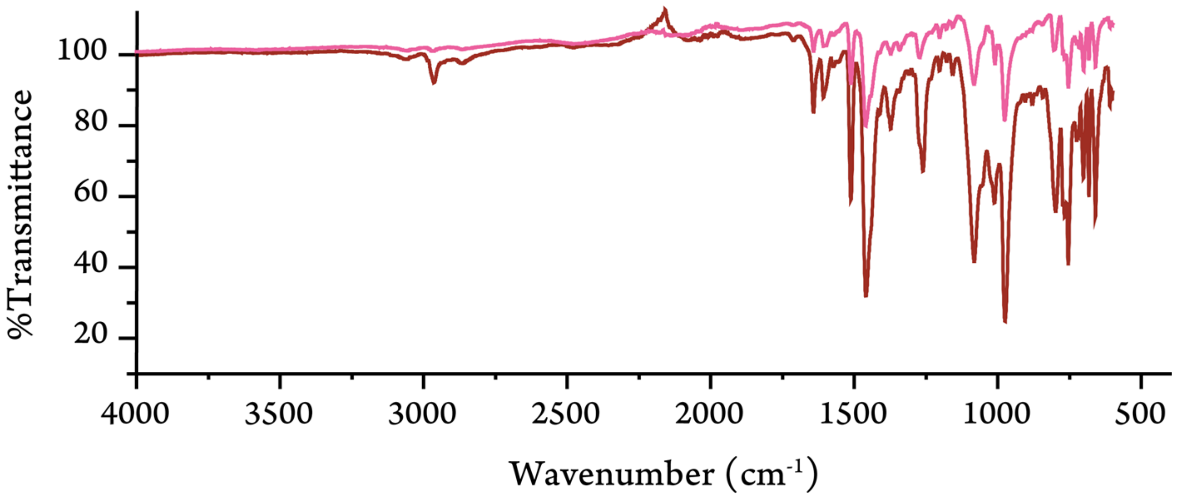

Figure S15. IR spectra of oxygenation products of [(TPP)Mn^{II}] with: 1 eq. of [(tmpa)Cu^{I}(MeCN)][$B(C_6F_5)_4$] (pink) or 2 eq. of [(tmpa)Cu^{I}(MeCN)][$B(C_6F_5)_4$] (maroon).

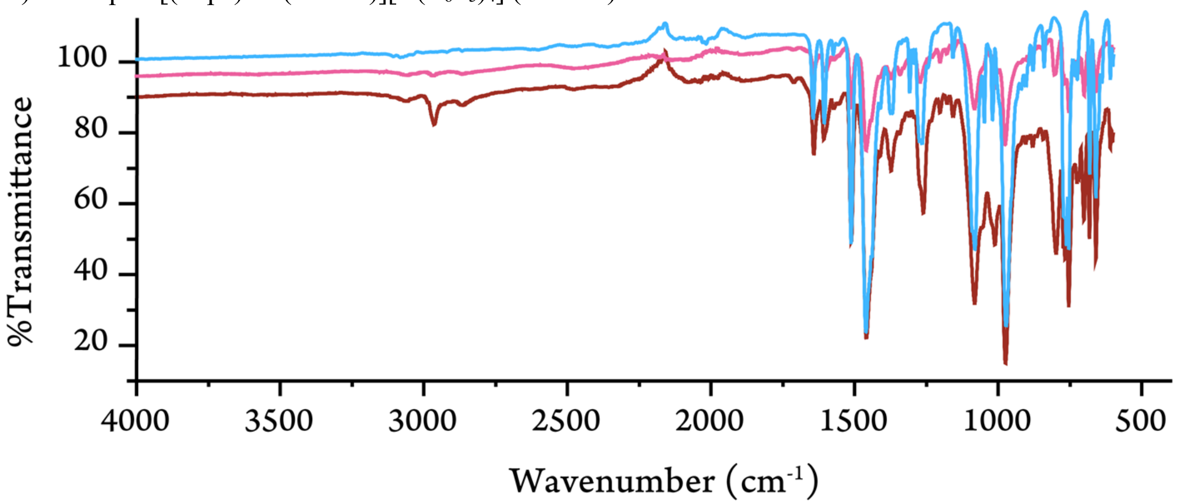

Figure S16. IR spectra of [(tmpa)Cu^{II}Cl][$B(C_6F_5)_4$] (blue) and the oxygenation products of [(TPP)Mn^{II}] with: 1 eq. of [(tmpa)Cu^{I}(MeCN)][$B(C_6F_5)_4$] (pink) or 2 eq. of [(tmpa)Cu^{I}(MeCN)][$B(C_6F_5)_4$] (maroon).

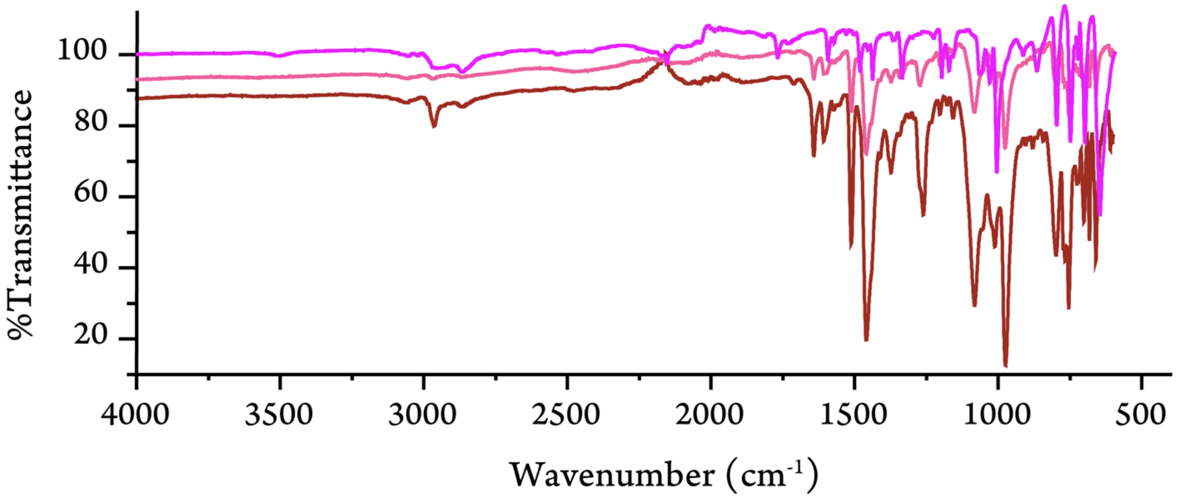

Figure S17. IR spectra of [(TPP)Mn^{III}(THF)$_2$]SbF_6 (magenta) and the oxygenation products of [(TPP)Mn^{II}] with: 1 eq. of [(tmpa)Cu^{I}(MeCN)][$B(C_6F_5)_4$] (pink) or 2 eq. of [(tmpa)Cu^{I}(MeCN)][$B(C_6F_5)_4$] (maroon).

Table S3. Metal-sensitive IR bands (cm^{-1}) of TPP manganese complexes.*

TPP	[(TPP)Mn^{II}]	[(TPP)Mn^{III}Cl]	[(TPP)Mn^{III}(THF)$_2$]SbF_6	1:1 eq. Product	1:2 eq. Product
1596	1594	1595	1598	1598	1598
1491	1473	1486	1487	1487 (sh)	1487 (sh)
1350	1333	1341	1340	1342	1342
1003	990	1006	1010	1010	1011
966	972	966 (sh)	971	(very broad)	(very broad)

* TPP data from Ref. [9]. TPP: Tetraphenylporphyrin. "1:1 eq. Product" and "1:2 eq. Product" refer to the oxygenation products of 1:1 and 1:2 eq. mixtures of [(TPP)Mn^{II}] and [(tmpa)Cu^{I}(MeCN)][$B(C_6F_5)_4$], respectively.

References

1. Hatano, K.; Anzai, K.; Iitaka, Y. The crystal and molecular structure of bis(methanol)-α,β,γ,δ-tetraphenylporphinatomanganese(III) perchlorate-methanol. A molecular structure relevant to the intermediate-spin six coordinate iron(III) porphyrin. *Bull. Chem. Soc. Jpn.* **1983**, *56*, 422-427, 10.1246/bcsj.56.422.
2. Hill, C.L.; Williamson, M.M. Structural and electronic properties of six-coordinate manganese(III) porphyrin cations. Crystal and molecular structure of bis(N,N-dimethylformamide)(tetraphenylporphinato)manganese(III) perchlorate, [Mn^{III} TPP(DMF)$_2$]$^+ClO_4^-$. *Inorg. Chem.* **1985**, *24*, 2836-2841, 10.1021/ic00212a027.
3. Hill, C.L.; Williamson, M.W. Electronic and structural properties of a reactive metalloporphyrin with N-oxide axial ligands. Crystal and molecular structure of bis(2,6-lutidine N-oxide)(tetraphenylporphinato)manganese(III) perchlorate. *Inorg. Chem.* **1985**, *24*, 3024-3030, 10.1021/ic00213a031.
4. Williamson, M.N.; Hill, C.L. Molecular stereochemistry of aquamanganese(III) porphyrins. Demonstrable effect of π-arene-porphyrin interaction in the metal coordination environment in the metalloporphyrin. *Inorg. Chem.* **1987**, *26*, 4155-4160, 10.1021/ic00272a005.

5. Scheidt, W.R.; Pearson, W.B.; Gosal, N. Structure of bis-(methanol)(meso-tetra-phenylporphinato)manganese(III) hexa-chloro-antimonate bis-(tetra-chloro-ethane) solvate. *Acta Cryst.* **1988**, *C44*, 927−929, 10.1107/S0108270187012411.
6. Fox, S.J.; Chen, L.; Khan, M.A.; Richter-Addo, G.B. Nitrosoarene complexes of manganese porphyrins. *Inorg. Chem.* **1997**, *36*, 6465-6467, 10.1021/ic970836b.
7. Bhyrappa, P.; Wilson, S.R.; Suslick, K.S. Hydrogen-bonded porphyrinic solids: Supramolecular networks of octahydroxy porphyrins. *J. Am. Chem. Soc.* **1997**, *119*, 8492-8502, 10.1021/ja971093w.
8. Tong, S.-l.; Zhang, J.; Yan, Y.; Hu, S.; Yu, J.; Yu, L. Self-assembled supramolecular architecture with alternating porphyrin and phthalocyanine, bonded by hydrogen bonding and π–π stacking. *Solid State Sci.* **2011**, *13*, 1967-1971, 10.1016/j.solidstatesciences.2011.08.026.
9. Kincaid, J.; Nakamoto, K. Vibrational spectra of transition metal complexes of tetraphenylporphine. *J. Inorg. Nucl. Chem.* **1975**, *37*, 85-89, 10.1016/0022-1902(75)80130-8.
